# Metabolic arsenal of giant viruses: Host hijack or self-use?

**DOI:** 10.7554/eLife.78674

**Published:** 2022-07-08

**Authors:** Djamal Brahim Belhaouari, Gabriel Augusto Pires De Souza, David C Lamb, Steven L Kelly, Jared V Goldstone, John J Stegeman, Philippe Colson, Bernard La Scola, Sarah Aherfi

**Affiliations:** 1 Microbes, Evolution, Phylogeny and Infection (MEPHI), UM63, Institut de Recherche pour le Développement (IRD), IHU Méditerranée Infection, Marseille, France, Aix-Marseille Université Marseille France; 2 Faculty of Medicine, Health and Life Sciences, Institute of Life Science, Swansea University Swansea United Kingdom; 3 Biology Department, Woods Hole Oceanographic Institution Woods Hole United States; 4 Assistance Publique - Hôpitaux de Marseille (AP-HM) Marseille France; Erasmus Medical Center Netherlands; Radboud University Medical Centre Netherlands

**Keywords:** giant viruses, primary metabolism, energy production

## Abstract

Viruses generally are defined as lacking the fundamental properties of living organisms in that they do not harbor an energy metabolism system or protein synthesis machinery. However, the discovery of giant viruses of amoeba has fundamentally challenged this view because of their exceptional genome properties, particle sizes and encoding of the enzyme machinery for some steps of protein synthesis. Although giant viruses are not able to replicate autonomously and still require a host for their multiplication, numerous metabolic genes involved in energy production have been recently detected in giant virus genomes from many environments. These findings have further blurred the boundaries that separate viruses and living organisms. Herein, we summarize information concerning genes and proteins involved in cellular metabolic pathways and their orthologues that have, surprisingly, been discovered in giant viruses. The remarkable diversity of metabolic genes described in giant viruses include genes encoding enzymes involved in glycolysis, gluconeogenesis, tricarboxylic acid cycle, photosynthesis, and β-oxidation. These viral genes are thought to have been acquired from diverse biological sources through lateral gene transfer early in the evolution of Nucleo-Cytoplasmic Large DNA Viruses, or in some cases more recently. It was assumed that viruses are capable of hijacking host metabolic networks. But the giant virus auxiliary metabolic genes also may represent another form of host metabolism manipulation, by expanding the catalytic capabilities of the host cells especially in harsh environments, providing the infected host cells with a selective evolutionary advantage compared to non-infected cells and hence favoring the viral replication. However, the mechanism of these genes' functionality remains unclear to date.

## Introduction

Viruses are the most abundant biological entities in the biosphere (Baltimore, 1971; Suttle, 2005; Suttle, 2007) and have been the subject of persistent debates as to their place in the domains of life. Active energy metabolism pathways and the autonomous ability to reproduce independently are the most fundamental criteria associated with living cells and therefore distinguish the three domains of life – archaea, bacteria, and eukarya – from viral entities. Specifically, the fundamental hallmark of all living organisms is the self-maintenance of their own functional and structural integrity via the biochemical transformations of environmental resources. (Dupré and O’Malley, 2009).

Viruses originally were defined as filterable agents capable of passing through membrane filters with pore sizes of 0.22 µm. Most viruses possess small genomes that carry only a handful of genes supporting their replication and capsid production in a host cell. Viruses have thus been characterized as ‘molecular genetic parasites’ that take advantage of cellular functions to replicate (Luria, 1959). Generally, once viruses find their replication site they hijack the host cellular metabolic pathways to their own advantage with the ultimate goal to replicate (Villarreal, 2005). However, ambiguity regarding the definition of viruses appears in the concept of a ‘virocell’, defined by Forterre, 2013 as a ‘living form’ of the virus, consisting of a living cell transformed to produce more virions (Forterre, 2013). At the blurred boundaries between viruses and cellular organisms, intracellular bacteria such as *Rickettsia* and *Chlamydia* also are known to hijack host energy metabolism enabling their persistence and proliferation (Driscoll et al., 2017; Gehre, 2015), a characteristic shared with viruses. Ultimately, however, viruses lack many of the essential attributes of living organisms, such as the ability to capture and store free energy, as well as autonomous replication and self-repairing capacity (Dupré and O’Malley, 2009; Regenmortel, 2010).

In 2003, the discovery of the giant viruses of amoeba revolutionized the view of viruses (La Scola et al., 2003). Specifically, the size of the giant virus particles (reaching up to 2.5 μm) and of their genome sizes (up to 2.5 Mb), encoding almost a thousand proteins, fundamentally changed the landscape of virus research (Philippe et al., 2013). Notably, *Acanthamoeba polyphaga* Mimivirus, the first giant virus of amoeba discovered, has a 1.2-Mbp genome, and possesses several genes encoding proteins involved in transcription and translation (Raoult et al., 2004; Raoult and Forterre, 2008b). Phylogenetic analyses have demonstrated that some bacterial and eukaryotic genes found in the Mimivirus genome, and other giant virus genomes, were acquired by horizontal gene transfer from amoebal hosts, and from amoebal bacteria parasites (Moreira and Brochier-Armanet, 2008), although most giant virus genes still have enigmatic origins.

Giant viruses represent a group of ancient viruses that are termed the Nucleo–Cytoplasmic Large DNA Viruses (NCLDVs). While some are found in amoebae, other of these viruses infect hosts ranging from algae to animals and form an apparently monophyletic group in the phylum *Nucleocytoviricota* (Colson et al., 2013). The giant viruses are classified into several families: *Mimiviridae*, *Pithoviridae*, *Pandoraviridae*, *Phycodnaviridae, Poxviridae, Iridoviridae, Asfarviridae,* and others. Many, including newly discovered viruses in the Mollivirus genus are not yet classified (Abergel et al., 2015; Legendre et al., 2014; Rolland et al., 2019).

The genomes of giant viruses encode proteins that had never before been identified in viruses. These proteins have sequence identity with closely related eukaryotic homologs (Chelkha et al., 2020; Sun et al., 2020). For example, *Cafeteria roenbergensis* virus, Klosneuviruses and Tupanviruses encode several gene products that are involved in protein translation (Abrahão et al., 2018; Schulz et al., 2017); the presence of such translation machinery had not been previously described in viruses. This finding suggested that those viruses may have relative independence from their hosts (and hence are ‘quasi-autonomous’ viruses) (Claverie and Abergel, 2010). This has led to a reconsidering of the historical parasitic hallmark of viruses (Abrahão et al., 2017; Raoult and Forterre, 2008a). In addition to the translation components, giant virus gene products have been implicated in RNA maturation, DNA maintenance, and proteostasis (Fischer et al., 2010; Yutin et al., 2013). Recently, defined metabolic genes involved in specialized cellular biochemical pathways have also been discovered in some giant viruses (Aherfi et al., 2022; Moniruzzaman et al., 2020; Schulz et al., 2020; Schvarcz and Steward, 2018). Herein, we analyze and describe giant virus genes encoding enzymes that are potentially involved in traditional biochemical pathways, findings that challenge the last distinguishing barriers separating the traditional domains of life and the giant viruses.

## Carbohydrate metabolism

Carbohydrates are key enzymatic substrates for many essential metabolic pathways in cells (Maughan, 2009). Generally, autotrophic organisms biosynthesize carbohydrates from carbon dioxide and water, via photosynthesis (Butterworth, 2005; Lehninger et al., 2004; Nelson and Cox, 2017). All organisms then break down stored or consumed carbohydrates in order to produce energy for cell functioning (Nelson and Cox, 2017). Both autotrophic and heterotrophic organisms temporarily store their generated energy in the form of high-energy molecules, for example adenosine triphosphate (ATP) and reduced nicotinamide adenine dinucleotide (NADH), for use in the various metabolic processes (Sanders, 2016).

Although some viruses in the *Nucleocytoviricota* phylum infect known hosts such as amoeba, it is not clear if these are actually the specific hosts. In any case, auxiliary metabolic genes (AMG) encoded by giant viruses appear to exercise control and modulation of host cellular energetic metabolism, especially during viral genome replication, gene expression, and virion assembly (Sun et al., 2020). Such control mechanisms can be based on transcriptional regulation of nuclear and mitochondrion-encoded genes that are involved in energy metabolism, as observed with coccolithoviruses (Ku et al., 2020). Single cell transcriptomics of the microalga *Emiliania huxleyi* during infection by its specific giant virus showed that either host or viral transcriptome dominated the generated mRNA pool, and that only very few cells were observed in intermediate states. The rapid shutdown of host transcript generation indicates that the *E. huxleyi* coccolithovirus massively takes over controlling synthesis of almost the entire mRNA pool.

**Figure 1.**
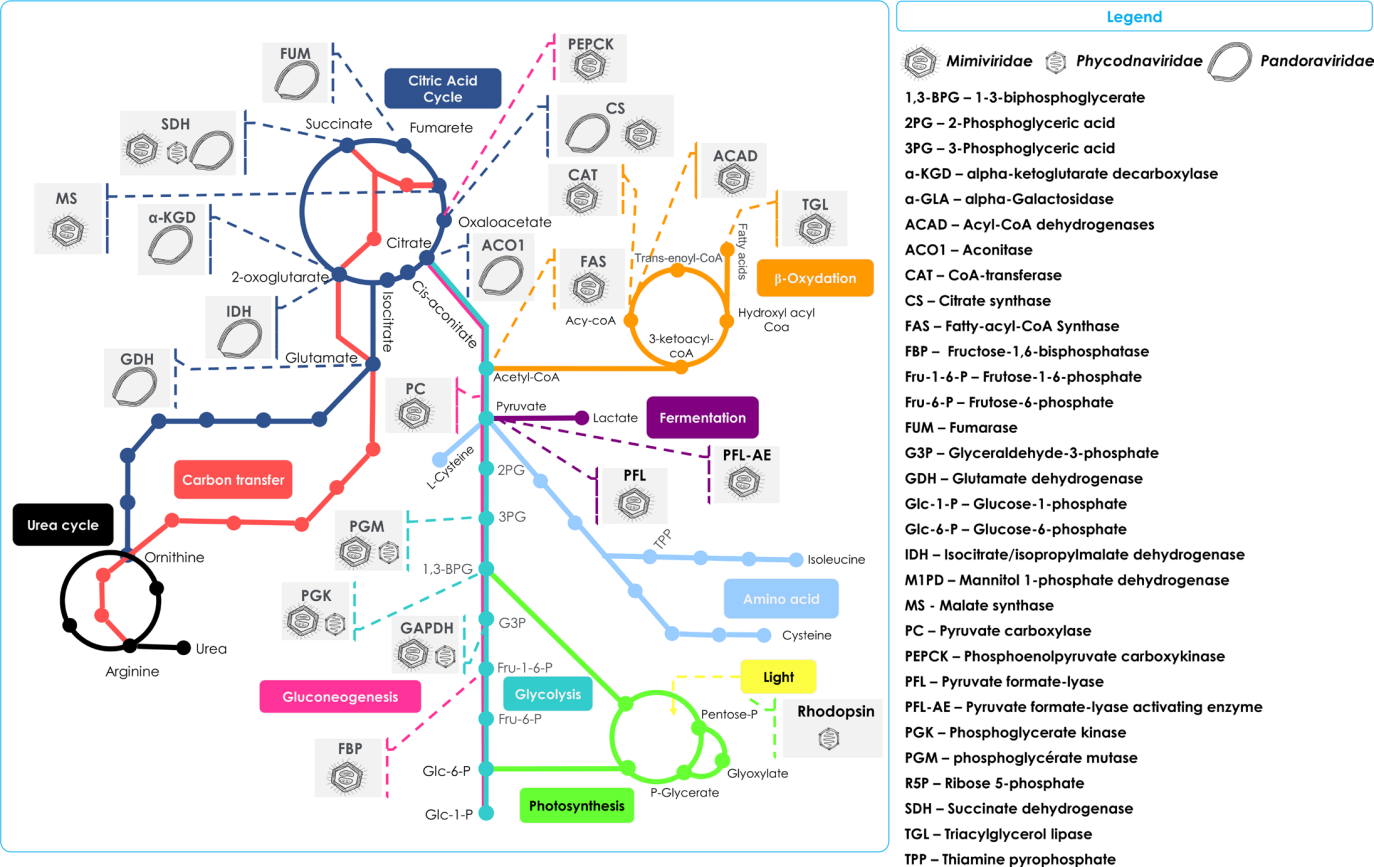
Schematic representation of the metabolic enzymes and pathways detected in NCLDVs. Schematic of the TCA cycle (dark blue) feeding into the Urea cycle (black); Carbon transfer (red); Gluconeogenesis (scarlet); Glycolysis (marine); Photosynthesis (green); Amino acid metabolism (blue); Fermentation (purple); and lipid β-oxidation (orange). Also shown in the Legend are the identified cellular enzymes and putative substrates which have been identified as being encoded in specific NCLDV genomes, here represented by *Mimiviridae*, *Phycodnaviridae,* and *Pandoraviridae*.

Many giant viral genes and gene products related to energy production and metabolic pathways have been identified recently (Aherfi et al., 2022; Ha et al., 2021; Moniruzzaman et al., 2020; Figure 1). In a detailed study examining a database of 2436 annotated *Nucleocytoviricota*, including metatranscriptomic data from California’s surface waters, hundreds of viruses, mainly from the *Mimiviridae* and *Phycodnaviridae* families, were identified. Many expressed viral transcripts in this dataset are genes involved in glycolysis (e.g. phosphofructokinase [EC 2.7.1.11], glyceraldehyde 3-phosphate dehydrogenase [EC 1.2. 1.12], phosphoglycerate mutase [EC 5.4.2.11]), gluconeogenesis (e.g. phosphoenolpyruvate carboxykinase [EC 4.1.1.32] and fructose-1,6-bisphosphatase [EC 3.1.3.11]), the tricarboxylic acid (TCA) cycle (e.g. succinate dehydrogenase [EC 1.3.5.1]), and the pentose phosphate pathway (e.g. 6-phosphogluconate dehydrogenase EC 1.1.1.1; Ha et al., 2021; Ha et al., 2021).

### Glycolysis and gluconeogenesis

Glycolysis is an ancient metabolic pathway that occurs in the cytoplasm of the cell and converts glucose into pyruvate, which is accompanied by transfer of electrons to NAD^+^ in order to generate NADH_2_ (Potter and Fothergill-Gilmore, 1993; Kumari, 2018). Inversely, gluconeogenesis is a process occurring in animals, fungi, plants, and bacteria, which results in the generation of glucose from non-carbohydrate carbon substrates such as lactate, amino acids, and glycerol (Rodwell et al., 2015).

**Figure 2.**
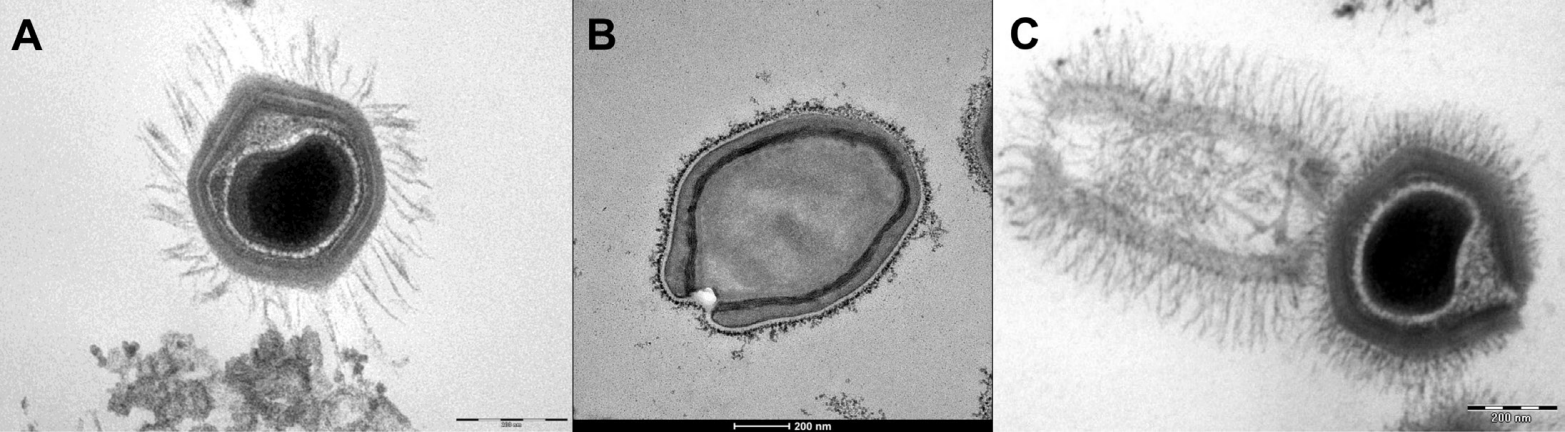
Transmission electron microscopy images of Mimivirus (**A**), *Pandoravirus massiliensis* (**B**) and Tupanvirus (**C**). (**A**) Mimivirus particle is composed of an external layer of dense fibers surrounding an icosahedral capsid and an internal membrane sac enveloping the virus genomic material. (**B**) *Pandoravirus massiliensis* virion is ovoid-shaped with an ostiole-like apex, measuring 1.0 μm in length and 0.5 μm in diameter. (**C**) Tupanvirus exhibits an icosahedral capsid similar to those of Mimivirus measuring ~450 nm. However, Tupanvirus virion harbors a large cylindrical tail (550 nm extension;~450 nm diameter, including fibrils) attached to the base of the capsid. Electron micrographs were acquired on a Tecnai G2 transmission electron microscope (Scale bar, 200 nm).

A genomic study using environmental metagenome-assembled genomes (MAGs), identified genes encoding the glycolytic enzymes glyceraldehyde-3-phosphate dehydrogenase, phosphoglycerate mutase, and phosphoglycerate kinase. These genes were considered to be particularly prevalent within the *Mimiviridae* (Figure 2A) and to a lesser extent in the *Phycodnaviridae* (Moniruzzaman et al., 2020). Studies of Mimivirus metagenomes identified a unique protein domain architecture, with a single fused gene encoding the enzymes glyceraldehyde-3-phosphate dehydrogenase and phosphoglycerate kinase, which serve in two adjacent steps in glycolysis (Moniruzzaman et al., 2020). Such protein architecture is unique thus far and may reflect a more efficient process for enzymatic activity. Although this possibility requires experimental verification, it nevertheless highlights that giant viruses may drive evolutionary innovations of their metabolic genes.

This same study revealed that three genes associated with gluconeogenesis were present in *Mimiviridae* genomes (Moniruzzaman et al., 2020): fructose 1,6-bisphosphatase, which catalyzes the biotransformation of fructose-1,6-bisphosphate into fructose-6-phosphate; phosphoenolpyruvate carboxykinase that converts oxaloacetate to phosphoenolpyruvate; and pyruvate carboxylase that catalyzes the carboxylation of pyruvate to form oxaloacetate (Figure 1, Table 1). The presence of such genes in infecting viruses also suggests there could be reprogramming of the host glycolysis pathways.

**Table 1.** List of metabolic enzymes detected in NCLDVs. The enzymes were grouped according to the metabolic pathway to which they belong and associated with the giant virus and/or the family in which they were identified.

Pathway	Enzyme	Function	KEGG*	Detected in	Family	Reference(s)
Amino acid catabolism	Glutamate dehydrogenase	Reversible conversion of glutamate to α-ketoglutarate and ammonia	R00243	Pandoravirus and others uncharacterized viruses†	Mimiviridae, Pandoraviridae and Phycodnaviridae	Moniruzzaman et al., 2020; Aherfi et al., 2018
	Glutamine synthetase	Condensation of glutamate and ammonia to form glutamine:	R00253	Uncharacterized viruses†	Mimiviridae	Ha et al., 2021
	Glutaminase	Hydrolysis of glutamine into glutamate	R00256	Uncharacterized viruses†	Mimiviridae	Moniruzzaman et al., 2020; Ha et al., 2021
Lipide catabolism and β-Oxydation	Triacylglycerol lipase	Degrades triacylglycerol into glycerol and fatty acids	R01369	Prymnesium kappa virus RF01	Mimiviridae	Blanc-Mathieu et al., 2021
	Fatty-acyl-CoA Synthase	Conversion of a acetyl-CoA and seven malonyl-CoA molecules to produce a Palmitoyl-CoA	R05190	Prymnesium kappa virus RF01	Mimiviridae	Blanc-Mathieu et al., 2021
	CoA-transferase	Conversion acyl-CoA and acetate into fatty acid anion and acetyl-CoA.	R00393	Prymnesium kappa virus RF01	Mimiviridae	Blanc-Mathieu et al., 2021
	Acyl-CoA dehydrogenase	Desaturation of the acyl-CoA esters	R00392	Prymnesium kappa virus RF01 and others uncharacterized viruses†	Mimiviridae	Blanc-Mathieu et al., 2021
Citric Acid Cycle	Succinate dehydrogenase	Conversion of succinate into fumarate	R02164	Prymnesium kappa virus RF01, Pandoravirus massiliensis and others uncharacterized viruses†	Mimiviridae, Pandoraviridae and Phycodnaviridae	Moniruzzaman et al., 2020; Aherfi et al., 2022; Blanc-Mathieu et al., 2021; Ha et al., 2021;
	Citrate synthase	Claisen condensation between acetyl CoA and oxaloacetate to yield, after hydrolysis of the thioester bond, citrate and CoA	R00351	Pandoravirus massiliensis and others uncharacterized viruses†	Pandoraviridae and Mimiviridae	Aherfi et al., 2022; Moniruzzaman et al., 2020
	Aconitase	Catalyzes the stereospecific isomerization of citrate to isocitrate via cis-aconitate in a non-redox reaction	R01324	Pandoravirus massiliensis and others uncharacterized viruses†	Pandoraviridae and Mimiviridae	Moniruzzaman et al., 2020; Rodrigues et al., 2019; Aherfi et al., 2022
	Isocitrate/isopropyl malate dehydrogenase	Oxidative decarboxylation of isocitrate, resulting in alpha-ketoglutarate and carbon dioxide.	R00267 / R01652	Pandoravirus massiliensis and others uncharacterized viruses†	Pandoraviridae and Mimiviridae	Aherfi et al., 2022; Moniruzzaman et al., 2020
	Malate synthase	Conversion of enzyme are acetyl-CoA, H2O, and glyoxylate into (S)-malate and CoA.	R00472	Uncharacterized viruses†	Mimiviridae	Ha et al., 2021
	Alpha-ketoglutarate decarboxylase	Conversion of α-ketoglutarate to succinyl-CoA and produces NADH directly providing electrons for the respiratory chain	R00272	Pandoravirus massiliensis	Pandoraviridae	Aherfi et al., 2022
	Fumarase	Conversion of fumarate to L-malate	R01082	Pandoravirus massiliensis	Pandoraviridae	Aherfi et al., 2022
Fermentation	Pyruvate formate-lyase	Catalyzes the reaction of pyruvate +CoA acetyl-CoA +formate	R00212	Tetraselmis virus	Phycodnaviridae	Müller et al., 2012; Schvarcz and Steward, 2018; Sun et al., 2020
	Formate-lyase activating enzyme	Converts pyruvate and CoA into acetyl CoA and formate	R04710	Tetraselmis virus	Phycodnaviridae	Müller et al., 2012; Schvarcz and Steward, 2018
Gluconeogenesis	Fructose bisphosphatase	Converts fructose-1,6-bisphosphate to fructose 6-phosphate	R00762	Uncharacterized viruses†	Mimiviridae	Moniruzzaman et al., 2020; Ha et al., 2021
	Phosphoenolpyruvate carboxykinase	Converts oxaloacetate into phosphoenolpyruvate and carbon dioxide.	R00341	Uncharacterized viruses†	Mimiviridae	Moniruzzaman et al., 2020; Ha et al., 2021
	Pyruvate carboxylase	Catalyzes the conversion of pyruvate to oxaloacetate	R00344	Uncharacterized viruses†	Mimiviridae	Moniruzzaman et al., 2020
Glycolysis	Glyceraldehyde-3-phosphate dehydrogenase	Conversion of pyruvate to oxaloacetate	R01061	Uncharacterized viruses†	Mimiviridae and Phycodnaviridae	Moniruzzaman et al., 2020
	Phosphoglycerate mutase	Transfers the phosphate from 3-phosphoglyceric acid (3 PG) to the second carbon to form 2-phosphoglyceric acid (2 PG)	R01518	Uncharacterized viruses†	Mimiviridae and Phycodnaviridae	Moniruzzaman et al., 2020; Ha et al., 2021
	Phosphoglycerate kinase	Catalyzes the formation of ATP from ADP and 1,3-diphosphoglycerate	R01512	Uncharacterized viruses†	Mimiviridae and Phycodnaviridae	Moniruzzaman et al., 2020
Photosynthesis	Rhodopsin	Generating a proton motive force across the cell membrane (light dependent)	R02903	Organic Lake Phycodnavirus 2 and Phaeocystis globosa virus	Phycodnaviridae	Needham et al., 2019; Yutin and Koonin, 2012; Schulz et al., 2020
Mannitol metabolism	Mannitol 1-phosphate dehydrogenase	Converts D-mannitol 1-phosphate and NAD +into fructose 6-phosphate, NADH and H+.	R00758	Tetraselmis virus	Phycodnaviridae	Schvarcz and Steward, 2018
Saccharide degradation	Alpha-galactosidase	Catalyzes the removal of terminal α-galactose groups from substrates such as glycoproteins and glycolipids	R01101	Tetraselmis virus	Phycodnaviridae	Schvarcz and Steward, 2018

* KEGG codes for the biochemical reactions described (https://www.genome.jp/kegg/reaction/).

† Enzymes detected in NCLDVs from metagenome-assembled genome analysis.

### Fermentation

Fermentation is a heterotrophic anaerobic redox process that uses an organic compound as the terminal electron acceptor (Müller et al., 2012; Zhou et al., 2017). It is considered as an ancient metabolic pathway. Genes encoding for two key fermentation genes – pyruvate formate-lyase and pyruvate formate-lyase activating enzyme – have been found in *Tetraselmis* virus (TetV) (*Chlorodendrephycae*) (Figure 1, Table 1), a mimivirus infecting the green algae *Tetraselmis* and land plants of the lineage *Viridiplantae* Müller et al., 2012. This viral host utilizes anaerobic energy metabolism in low-oxygen conditions, suggesting horizontal gene transfer (Müller et al., 2012; Schvarcz and Steward, 2018; T.-W. Sun et al., 2020, p. 1). Pyruvate formate-lyase catalyzes the reversible conversion of pyruvate and coenzyme-A into formate and acetyl-CoA (Knappe et al., 1974). It is thought that in low oxygen conditions the host may use these viral genes to generate the required energy via manipulation of anaerobic energy metabolism. In the absence of light, algae and bacteria deplete dissolved oxygen in the superficial water. In anoxic conditions, the fermentation process thus may favor viral spreading, with the metabolism of infected cells being potentially maintained by virally encoded fermentation genes, an advantage for the viral host (Schvarcz and Steward, 2018).

Other putative viral fermentation genes that have been identified include the mannitol metabolism protein, mannitol 1-phosphate dehydrogenase and the saccharide degradation enzyme alpha-galactosidase in the *Tetraselmis* virus (Table 1). However, the role of these genes in the virus is less clear (Schvarcz and Steward, 2018).

### Tricarboxylic acid cycle

The tricarboxylic acid (TCA) cycle – also known as the Krebs cycle – is a linked series of biochemical reactions used by all aerobic organisms to release stored energy via the oxidation of acetyl-CoA, derived from carbohydrates, fats, and proteins (Smith and Morowitz, 2004). It is the central metabolic hub for many biochemical pathways. It begins from acetate (in the form of acetyl-CoA) and water, to reduce NAD +into NADH, with the concomitant release of carbon dioxide (Krebs and Johnson, 1937; Meléndez-Hevia et al., 1996; Noor et al., 2010). The NADH generated by this cycle enters the oxidative phosphorylation (electron transport) pathway. As a result of these two closely linked metabolic pathways, several molecules of ATP are produced.

In the previously cited study based on MAGs, predicted TCA-related gene products were the most represented, especially aconitase and succinate dehydrogenase, encoded in *Mimiviridae* and *Phycodnaviridae* genomes (Moniruzzaman et al., 2020). Aconitase was previously reported in Tupanvirus as well (Rodrigues et al., 2019; Figures 1 and 2.C Table 1). In cells, aconitase catalyzes the stereospecific isomerization of citrate to isocitrate via *cis*-aconitate in a non-redox reaction. Succinate dehydrogenase is a complex enzyme that catalyzes the conversion of succinate into fumarate, which generates electrons used to reduce oxygen into water (Oyedotun and Lemire, 2004). The viral TCA enzymes may be utilized to boost host TCA cycle steps and hence energy production, possibly providing competitive advantages to the host.

The alga-infecting giant virus *Prymnesium* kappa virus RF01, which belongs to the *Mimiviridae* family, harbors six energy metabolism gene homologs (Blanc-Mathieu et al., 2021). Among them, there are the four succinate dehydrogenase subunits (A-D), an enzyme involved in oxidative phosphorylation pathway and the TCA cycle (Figure 1, Table 1). The gene encoding subunit A of this complex protein is transcribed during infection, again intimating that host energy metabolism could be modulated by the virus (Blanc-Mathieu et al., 2021). Putative succinate dehydrogenase genes detected by metatranscriptomic analysis were associated with *Mimiviridae,* suggesting the gene product to be highly expressed in the virosphere of surface oceanic waters of California (USA) (Ha et al., 2021), and may be widespread among marine *Mimiviridae* (Blanc-Mathieu et al., 2021). In the same study, malate synthase and isocitrate lyase, other key enzymes of the TCA cycle, were found expressed in different time points in viruses in the *Mimiviridae* (Ha et al., 2021; Figure 1, Table 1).

Several predicted proteins recently found in *Pandoravirus massiliensis* (Figure 2B) are homologues of enzymes involved in the TCA cycle, albeit with low % identities: citrate synthase, aconitase, isocitrate/isopropyl malate dehydrogenase, alpha-ketoglutarate decarboxylase, succinate dehydrogenase, and fumarase (Figure 1, Table 1). The predicted isocitrate dehydrogenase of *P. massiliensis* was functional both in crude viral particles and following in vitro reconstitution of the purified recombinant protein. In addition, it was also demonstrated experimentally that *P. massiliensis* can generate an electrochemical gradient, an essential component of all living cells (Aherfi et al., 2022). The membrane voltage in *P. massiliensis* may be involved in the amoeba infection process, notably in the early stages of infection (Aherfi et al., 2022), helping the virus to release its DNA into host cells. Besides, it has been shown previously that the inhibition of potassium ion channels of *Chlorella* viruses, proteins related to membrane voltage, causes the depolarization of host cells and consequently the inhibition of viral DNA release into the host cell (Frohns et al., 2006; Neupärtl et al., 2008). Whether similar mechanisms are involved in *P. massiliensis* is unknown.

These findings potentially suggest that these viruses use such genes either to produce energy autonomously, or to stimulate the host energy metabolism in the aim to confer a replicative advantage to infected cells. Further investigations such as expression of these genes, followed by enzymatic activity assays are needed for a detailed comprehension of these enzymes.

## Lipids

Lipid metabolism consists of the catabolism of fatty acids and steroids/sterols in order to generate energy, and anabolic processes used to synthesize new lipids from smaller constituent molecules. Generally, lipids classified as fatty acids, triacylglycerols, phospholipids, sterols and sphingolipids are utilized in energy generation. Lipidomic β-oxidation is the main metabolic pathway for the degradation of fatty acid molecules to generate acetyl-CoA, which then enters in the TCA cycle.

A recent metatranscriptomic time-series study from the California Current surface waters has identified many novel transcripts putatively for lipid metabolism enzymes in viruses in the *Nucleocytoviricota*. Identified viral genes included those predicted to be involved in lipid β-oxidation (Ha et al., 2021). Genes encoding acyl-CoA dehydrogenases (EC 1.3.8.7), which catalyze the first committed enzymatic step of lipid β-oxidation, were consistently expressed across sampled timepoints, especially in the Mimiviridae. This finding again suggests that virus-mediated reprogramming of host central carbon metabolism is functional and occurs in oceanic surface waters (Ha et al., 2021).

In Prymnesium kappa virus RF01 (PCV RF01), numerous genes were found that are predicted to encode enzymes known to be involved in cellular lipid metabolism, such as a triacylglycerol lipase, which degrades triacylglycerol into glycerol and fatty acids (Figure 1, Table 1). These metabolites are used as precursors for ATP production via glycolysis and β-oxidation, respectively. In addition, it has been found that PCV RF01 encodes the key β-oxidation enzymes fatty acyl-CoA synthetase (EC 6.2.1.1), CoA-transferase (EC 2.8.3.8), and acyl-CoA dehydrogenase (EC 1.3.8.7) (Blanc-Mathieu et al., 2021). However, enzymes involved in the two intermediate steps immediately following each oxidation, enoyl-CoA hydratase (EC 4.2.1.17) or a β-ketothiolase (EC2.3.1.16), have not been detected in any giant virus to-date.

## Amino-acids / proteins

In cells, protein and/or amino acid utilization as a source of energy release mainly occurs when the intake of carbohydrates or lipids is insufficient to supply the required energy demand (Hothersall and Ahmed, 2013). In prokaryotes, and in mitochondria, glutamate dehydrogenase (EC 1.4.1.2) catalyzes the reversible conversion of glutamate to α-ketoglutarate and ammonia while reducing NAD(P)+to NAD(P)H. The α-ketoglutarate is consumed by the TCA cycle to produce ATP (Glevarec et al., 2004; Miller and Magasanik, 1990; Plaitakis et al., 2017). MAGs studies of members of multiple NCLDV families report the presence of putative glutamate dehydrogenase, glutamine synthase (EC 6.3.1.2), and glutaminase (EC 3.5.1.2) enzymes (Moniruzzaman et al., 2020). Other analyses confirmed the presence of glutamate dehydrogenase in members of the *Pandoraviridae* family (Figure 1, Table 1; Aherfi et al., 2018; Hosokawa et al., 2021; Legendre et al., 2018; Philippe et al., 2013).

Glutaminase and glutamine synthase, two cellular enzymes that regulate primary energy metabolism, found in *Mimiviridae* were expressed upon infection (Ha et al., 2021). The glutaminase catalyzes the hydrolysis of glutamine into glutamate, regulating cellular energy metabolism via the increase of glutamate and α-ketoglutarate. This results in enhanced mitochondrial respiration and ATP generation (Campos-Sandoval et al., 2007; Hu et al., 2010). Glutamine synthetase plays a key role in the cellular utilization of carbon and nitrogen sources, helps modulate the energy budget in bacteria and mediates the release of energy stored in glutamine, that increases especially in stressed cells (Aldarini et al., 2017). Thus, the presence of such glutaminolysis enzymes in giant viruses may help reprogram metabolism by maximizing glutamine catabolism and thus increase available energy to promote virus replication and virion production (Ha et al., 2021).

## Energy from inorganic compounds including photosynthesis

### Energy from inorganic compounds

Chemolithotrophy is a metabolic process whereby energy is derived from the oxidation of inorganic compounds such as hydrogen (Friedrich and Schwartz, 1993), reduced sulfur compounds (Friedrich, 1997), hydrogen sulfide, thiosulfate, ferrous iron, and ammonia (Jetten et al., 1998). Microbial oxidation of inorganic compounds is governed by chemical and enzymatic reactions to generate energy (ATP) and reducing power (NADH).

The most common chemotrophic organisms that oxidize inorganic compounds are prokaryotic. Nonetheless, diverse deep-sea viruses including members of the *Podoviridae, Siphoviridae*, and *Myoviridae,* have been reported to contain genes putatively encoding the α and γ subunits of the reverse-acting dissimilatory sulfite reductase (Rdsr), an enzyme that oxidizes the element sulfur. It is assumed that this gene is used to maintain or augment host cellular processes during infection and to redirect energy and resources towards viral production (Anantharaman et al., 2014).

A recent metagenomic study found that predicted ferric reductase enzymes were encoded in several NCLDV genomes (Schulz et al., 2020). In cellular organisms, ferric reductases function as a terminal reductase in an electron transport chain, by reducing ferric ion Fe^3+^ into ferrous ion Fe^2+^. Ferric reductase enzymes are also critical for the assimilatory iron pathway in organisms (Lovley, 2002; Lovley and Coates, 2000; Lovley et al., 1998). The reduction of ferric iron combined with a proton gradient through the cell membrane, is used by membrane-bound ATP synthase to generate the ATP (Schröder et al., 2003). This proton gradient can also be used to reduce NAD(P)^+^ in chemolithotrophs for several biosynthetic reactions (Lovley, 2002; Lovley and Coates, 2000). The presence of these enzymes in giant viruses and their functioning biochemistry may support infected host cell(s) metabolism and confer on them a competitive advantage in suboxic environments (Márquez et al., 2007). Moreover, these enzymes may also play a significant role in modifying the composition of the surrounding chemical environment, which can impact other microorganisms, most notably those that use iron respiration (Schulz et al., 2020).

### Photosynthesis

Photosynthesis is arguably the most important biological process functioning in nature, and is responsible for the existence of most life on Earth. It is essential for producing and maintaining the oxygen content of the atmosphere and supplies most of the energy necessary for life on the planet (Bryant and Frigaard, 2006). Photosynthesis is a pivotal process used by many autotrophic organisms, enabling them to convert light energy into chemical energy which is stored in the form of carbohydrates synthesized from carbon dioxide and water (Blankenship, 2010; Olson, 2006). Subsequently, the carbohydrates can be released to fuel the organism’s cellular metabolic activities. However, viral infections can potentially affect and/or redirect metabolic pathways in photosynthetic host organisms. For example, infection with a mimivirus has been shown to suppress transcripts related to photosynthesis as well as cytoskeleton formation in brown-tide forming pelagophyte *Aureococcus anophagefferens* (Moniruzzaman et al., 2018; see below).

In marine environments, viruses are the most abundant biological entities and drive organism population control by consistently impacting nutrient and biogeochemical cycling in these ecosystems (Hurwitz et al., 2013; Juneau et al., 2003; Suttle, 2016; Villarreal and Witzany, 2010). Marine viruses also affect algal blooms and their diversity as well as species distribution (Juneau et al., 2003). Marine viral genes that encode for numerous proteins involved in photosynthetic biology have already been found and characterized, especially in cyanophages (Alperovitch-Lavy et al., 2011; Coutinho et al., 2017; Lindell et al., 2005; Sharon et al., 2009; Sullivan et al., 2006). These proteins include photosystems I and II that drive the complete photosynthetic process during phage infection, with the overall effect of promoting maximal phage replication (Fridman et al., 2017).

Most NCLDV groups appear to be present ubiquitously in marine environments, based on metagenomics (Endo et al., 2020). The majority of these are closely related to the families *Mimiviridae* and *Phycodnaviridae*: of the more than 6,700 polymerase B genes (PolB; a conserved marker of NCLDVs) assembled from the Tara Oceans dataset, 5091 were related to *Mimiviridae* and 981 to *Phycodnaviridae* (Endo et al., 2020). However, other studies suggest that certain NCLDVs may be endemic to the regions in which they were identified (Needham et al., 2019), and there is considerable undiscovered diversity in marine giant viruses.

Many genes detected in these reassembled NCLDV genomes were predicted to encode for proteins with putative roles in photosynthesis, suggesting that reprogramming host metabolism may be a common phenomenon employed by NCLDVs (Schulz et al., 2020). Giant viruses thus may fundamentally impact photosynthetic processes in marine and freshwater protists (Needham et al., 2019; Endo et al., 2020; Moniruzzaman et al., 2017; Short, 2012). However, that NCLDV apparently endogenize into various green algae, so the metagenome assemblies may not be accurate in this case (Moniruzzaman et al., 2020).

Another example of the biochemical consequences of viral infections in marine ecosystems is represented by a recent transcriptome study, providing new insights regarding transcriptional remodeling in *Aureococcus* cells when infected by the giant virus *Aureococcus anophagefferens*. Immediately after *A. anophagefferens* infection, *Aureococcus* cellular genes related to light harvesting, photosystem structure, and isoprenoid biosynthesis are down-regulated while porphyrin biosynthesis genes are up-regulated (Moniruzzaman et al., 2018). This over-expression, by increasing intracellular porphyrin concentrations in infected cells, may increase cellular oxidative stress and may represent a host defense mechanism against the viral attack.

The choanoviruses in the phylum *Nucleocytoviricota* parasitize choanoflagellates, protistan predators. Choanovirus genomes assembled from metagenomic sequences encode the complete rhodopsin-based photosystem (Needham et al., 2019). This virally-induced light-driven energy transfer is closely connected with host ATP synthases and elegantly illustrates how giant viruses can modulate and alter nutrition and use of organic compounds by their unicellular eukaryotic hosts in marine environments (Needham et al., 2019). The photosystem genes in the choanoviruses may be the result of unique (or multiple) horizontal gene transfers events (Needham et al., 2019).

In another example of viral manipulation of host primary metabolism, genes encoding for proteorhodopsin were identified in the genomes of NCLDV Organic Lake Phycodnavirus (OLPV) 2 and *Phaeocystis globosa* virus (PGV) (Yutin and Koonin, 2012). Proteorhodopsins are photoreceptors found in marine planktonic bacteria, archaea, and eukaryotes (Béjà et al., 2000; Buhr et al., 2015; Frigaard et al., 2006; Lin et al., 2010; Slamovits et al., 2011). Metagenomic studies of extended sampling of NCLDV genomes revealed further that NCLDVs encode a large panel of diverse rhodopsins, representing one quarter of the current total diversity of rhodopsin proteins known to date (Schulz et al., 2020). Their role as sensory rhodopsins may complement the host’s rhodopsin function, or conversely confer a new functionality to the host. It has been suggested that their expression may induce host phototaxis, and stimulate host relocation to nutrient-rich areas that are necessary for viral replication (Yutin and Koonin, 2012). The origins of viral proteorhodopsin genes remain unknown, but they may have been captured from their unicellular eukaryotic hosts. Phylogenetic analyses based on sequences retrieved from metagenomic data argue for a monophyletic group formed by NCLDV rhodopsins, suggesting that these genes represent an ancestral trait shared by these viruses and which were then subsequently lost (Schulz et al., 2020). Furthermore, genes encoding putative carotenoid oxygenases have also been detected in these metagenomes. Viral carotenoid oxygenases may have the ability to modulate the host’s capacity to capture light and/or synthesize bioactive compounds. Thus, they may act in association with rhodopsins in order to provide metabolic advantages to infected populations (Schulz et al., 2020).

## Others

In addition to primary metabolism energetic enzymes described above, a remarkable diversity of auxiliary metabolic genes involved in (i) carbon metabolism, nitrogen and nutrient cycling and (ii) soil organic matter degradation, have been found in marine viral communities (Table 2). It is hypothesized that these metabolic genes can complement deficient host metabolic pathways in order to sustain their host under environmental stressful conditions, with the overall goal to increase or maintain viral replication (Coutinho et al., 2017; Howard-Varona et al., 2020; Hurwitz and U’Ren, 2016). Such host / virus interactions appear to play an important role in the global ecosystem (Brum and Sullivan, 2015; Hurwitz and U’Ren, 2016; Suttle, 2007; Zimmerman et al., 2020).

**Table 2.** List of enzymes with other biological roles detected in NCLDVs. The enzymes were grouped according to the biological process to which they belong and associated with the giant virus and/or the family in which they were identified.

Biological process	Enzyme	Function	Detected in	Family
Oxidative stress regulation	Superoxide dismutase	Catalyzes the dismutation of the superoxide radical into ordinary molecular oxygen and hydrogen	Emiliania huxleyi virus, Megavirus chiliensis, and others uncharacterized viruses*	*Mimiviridae* and *Phycodnaviridae*
	Glutathione peroxidase	Reduces free hydrogen peroxide to water.	Emiliania huxleyi virus and others uncharacterized viruses*	*Mimiviridae* and *Phycodnaviridae*
Ion’s transport and assimilation	Ammonium transporter	Mediates the transport of ammonium ions	Ostreococcus virus 6	*Phycodnaviridae*
	Phosphate transporter	Mediates the transport of phosphate ions	Uncharacterized viruses	*Mimiviridae* and *Phycodnaviridae*
	Sulfur transporter	Mediates the transport of sulfur ions	Uncharacterized viruses	*Mimiviridae* and *Phycodnaviridae*
	Magnesium transporter	Mediates the transport of magnesium ions	Uncharacterized viruses	*Mimiviridae* and *Phycodnaviridae*
	Iron transporter	Mediates the transport of iron ions	Uncharacterized viruses	*Mimiviridae* and *Phycodnaviridae*
	Ferritin	Iron storage protein	Uncharacterized viruses	*Mimiviridae* and *Phycodnaviridae*
	Ferric reductases	Oxidation of NADPH and transference the electron to reduce metals like iron and copper	Uncharacterized viruses	*Mimiviridae* and *Phycodnaviridae*
	Multicopper oxidases	Oxidation of different substrates by accepting electrons at a mononuclear copper centre and transferring them to a trinuclear copper centre.	Uncharacterized viruses	*Mimiviridae* and *Phycodnaviridae*
Biosynthesis of glycosphingolipids	Serine palmitoyltransferase	Catalyzes the decarboxylative condensation of L-serine and palmitoyl coenzyme A to 3-ketodihydrosphingosine.	Coccolitho virus	*Phycodnaviridae*
Polysaccharide biosynthesis	Hyaluronan synthase	Produces the glycosaminoglycan hyaluronan from UDP-α-N-acetyl-D-glucosamine and UDP-α-D-glucuronate	Chlorovirus CVK2	*Phycodnaviridae*
	Chitin synthase	Produces Uridine diphosphate (UDP) and [[[1,4-(N-acetyl-beta-D-glucosaminyl)]n+1]] from UDP-GlcNAc and [[[1,4-(N-acetyl-beta-D-glucosaminyl)]n]]	Chlorovirus CVK2	*Phycodnaviridae*
Sugar metabolism	GDP-D-mannose 4,6 dehydratase	Conversion of GDP-(d)-mannose to GDP-4-keto, 6-deoxy-(d)-mannose	Paramecium bursaria Chlorella virus 1	*Phycodnaviridae*
	GDP-4-keto-6-deoxy-D-mannose epimerase/reductase	Converts GDP-4-keto-6-deoxy-d-mannose into GDP-l-fucose	Paramecium bursaria Chlorella virus 1	*Phycodnaviridae*
Polysaccharides degradation	Chitinase	Chitin degradation by cleaves the disaccharide to its monomer subunits	Chlorella virus PBCV-1	*Phycodnaviridae*
	1–3-beta glucanase	Successive hydrolysis at the nonreducing end of the glucan, resulting in the formation of oligosaccharides and glucose	Chlorella virus PBCV-1	*Phycodnaviridae*
	Pectate lyase	Randomly cleaves α–1,4-polygalacturonic acid via a β-elimination reaction	Aureococcus anophagefferens virus	*Phycodnaviridae*

* Enzyme’s genes were detected in NCLDVs from metagenome-assembled genome analysis.

Other gene products have been implicated in the reprograming of cellular physiology during giant virus infection, especially in nutrient processing and oxidative stress (Monier et al., 2017; Moniruzzaman et al., 2018; Sheyn et al., 2016). For example, it has been reported that *Mimiviridae* and *Phycodnaviridae* families exhibit superoxide dismutase and glutathione peroxidase activities encoded by crucial enzymes involved in oxidative stress regulation (Table 2). A superoxide dismutase encoding gene, involved in the breakdown of reactive oxygen species (ROS), was found encoded in the *Megavirus chiliensis* genome (Lartigue et al., 2015). These enzymes likely help to protect the viral replication machinery from damage by ROS generated during viral infection (Moniruzzaman et al., 2018; Moniruzzaman et al., 2020).

Sheyn et al., 2016. have shown that during lytic infection by *Emiliana huxleyi* virus, which infects the cosmopolitan unicellular eukaryotic algal host *Emiliania huxleyi*, glutathione was overproduced and that hydrogen peroxide (H_2_O_2_) was the major ROS during the onset of the lytic phase of infection. Moreover, the concomitant production of GSH and H_2_O_2_ occurred in the same cellular subpopulations that exhibited a higher rate of infection compared with cells that had little or no GSH and H_2_O_2_. Interestingly, the inhibition of ROS production significantly reduced virion production and inhibited host cell death (Sheyn et al., 2016).

*Ostreococcus virus 6*, a virus of the green alga *Ostreococcus tauri*, harbors a gene encoding an ammonium transporter that is expressed during viral infection. The activity of this protein may increase host NH4 +uptake rates to fulfill the increased nitrogen requirements of infected cells undergoing viral replication (Monier et al., 2017). Moreover, numerous genes that encode for phosphate, sulfur, magnesium and iron transporters, ferric reductases and multicopper oxidases were identified recently in several NCLDV clades (Moniruzzaman et al., 2020; Schulz et al., 2017). These genes may boost the acquisition of these essential nutrients for host cell metabolism during virion production, notably in marine environments where nutrient availability may be limiting for cellular growth (Behrenfeld and Kolber, 1999; Herbik et al., 2002; Hogle et al., 2018; Saikia et al., 2014).

In addition to enzymes implicated in carbohydrate metabolism for energy generation as described above, some giant virus genes also encode proteins involved in the biosynthesis and manipulation of carbohydrate, lipid, and nucleotide metabolism. *Coccolithoviruses*, belonging to the family *Phycodnaviridae*, possess a cluster of biosynthetic genes, including a serine palmitoyltransferase encoding gene, involved in the biosynthesis of glycosphingolipids, a major component of the virion membrane envelopes. High glycosphingolipid producing strains of *E. huxleyi* virus (EhV), which are extremely virulent and harbor a greater infectivity at high host densities, provide a selective advantage under laboratory conditions. However, field data obtained from natural environments suggest a better survival rate of slow glycosphingolipid producing EhVs, where lower host densities are encountered. Viral glycosphingolipid biosynthesis impacts on ecological balance in natural oceanic environments, where *E. huxleyi* plays an essential role in the global carbon cycle (Nissimov et al., 2019). Moreover, it has been shown that viral glycosphingolipids are able to suppress host cell growth by inducing programmed cell death (Monier et al., 2009; Rosenwasser et al., 2016; Vardi et al., 2009; Wilson et al., 2005).

Chloroviruses encode enzymes involved in (i) polysaccharide biosynthesis for example hyaluronan synthase, chitin synthase (Mohammed Ali et al., 2005). (b); (ii) sugar metabolism, for example GDP-D-mannose 4,6 dehydratase and GDP-4-keto-6-deoxy-D-mannose epimerase/reductase (Tonetti et al., 2003); and (iii) polysaccharides degradation, for example chitinase and 1–3-beta glucanase (Sun et al., 1999; Sun et al., 2000). These enzymes may be necessary for the infection cycle, for the ability to enter and exit the host cell (Van Etten et al., 2017). Polysaccharide lyases for example*,* pectate lyase, were also found to be encoded in the *A. anophagefferens* virus (AaV) genome (Moniruzzaman et al., 2014). It is probable that the genes encoding these putative enzymes were acquired by lateral gene transfer from either bacteria or from their host *A. anophagefferens* (Moniruzzaman et al., 2014). AaV genes encoding polysaccharide lyases have been mainly detected in ocean sampling throughout the world suggesting that these viruses play important role(s) in shaping the biogeochemical potential, in global marine system communities (Gann et al., 2020).

Analysis of the *A. polyphaga* Mimivirus genome revealed the presence of genes potentially involved in the biosynthesis of viosamine, which may have a role in the formation of the long fibers surrounding the virions (Piacente et al., 2012). Moreover, several giant viruses harbor the metabolic machinery that is involved in the production of glycoconjugate substrates (Fischer et al., 2010; Philippe et al., 2013; Piacente et al., 2015; Santini et al., 2013). Such genes include those encoding for nucleotide sugars enzymes and glycosyltransferases (Piacente et al., 2014). These viral enzymes may play structural role(s), which seems obvious when considering the highly glycosylated surface of the virions that protect them from the environment. They also may play a role in the phagocytic vacuole (Lairson et al., 2008; Legendre et al., 2014; Legendre et al., 2015; Piacente et al., 2014).

Finally, even steroid metabolism may be manipulated by NCLDV infection. Genomes of many mimiviruses and pandoraviruses contain cytochrome P450 genes, which encode P450 monooxygenase enzymes (Lamb et al., 2019). P450s are key enzymes in the metabolism of numerous endogenous regulatory molecules and xenobiotics in *Bacteria, Archaea,* and *Eukarya*. To support this suggestion, multiple genes involved in other aspects of steroid metabolism, notably steroid reductases, are also present and expressed (typically early) during the course of NCLDV infection (De Souza et al., 2021).

### Conclusion

Previously, viruses were traditionally defined as molecular genetic parasites, accessories to cellular life, and lacking many of the essential criteria that define living organisms, such as the ability to capture and store free energy. However, this strict paradigm has now been fundamentally challenged by the identification of a large number of energy-linked metabolic genes encoded in some NCLDV genomes, with sequence identity to cellular orthologs, including those possibly involved in energy generation from organic and inorganic compounds. These genes are thought to have been acquired by NCLDV from diverse sources, and especially from their hosts through lateral gene transfer. Notably, a number of the genes considered here were identified in metagenomic studies, which raises a possibility that some of these genes inferred as being in viral genomes might instead represent genes from bacteria, and emphasize the need for viromics benchmarking (Pratama et al., 2021). Indeed, the filtration process to discard non-viral sequences does not completely exclude the possibility of a contamination. Recognizing that caveat, we expect that sequences analysis in such metagenomic studies cited here employed benchmarking and curation approaches sufficient to discriminate between viral and microbial sequences. However, it should be noted that NCLDV apparently endogenize into various green algae, complicating the accurate assignment of partial metagenome assemblies, but also emphasizing the role of HGT in host and virus evolution (Moniruzzaman et al., 2020). Isolation of these viruses by culture and direct sequencing of viral strains combined with further analysis in molecular biology and biochemistry should be a complementary approach to elucidate their presence in the viral genomes.

Phylogenetic analysis reveals that these metabolic genes have diversified into virus-specific lineages. However, the origin of many of these genes still remains obscure, particularly as these NCLDV metabolism sequences tend to cluster together phylogenetically in deep-branching clades. This finding suggests that they diverged from their cellular orthologs in the distant past (Aherfi et al., 2022; Moniruzzaman et al., 2020; Needham et al., 2019; Schvarcz and Steward, 2018). These complex metabolic genes are thought to play a role(s) in manipulating host energy metabolism pathways during infection and help to ensure optimal intracellular host environments required for viral replication. Indeed, viruses are able to reshape the virocell metabolism not only by reprogramming host-encoded metabolic networks, but also by expanding the virocell metabolic abilities / needs by introducing new viral encoded auxiliary metabolic genes. This concept is particularly well illustrated by marine viruses, which play key role(s) in ecology, biogeochemistry and evolution of the marine environment, by impacting nutrient recycling and driving species composition. The massive amounts of viral macromolecules and viral particles that are synthesized in an infected cell impose heavy demands on the host. Biosynthesis of the elements that make up a viral particle, namely nucleotides, amino acids and sometimes fatty acids, requires energy in the form of ATP. The recruitment of cellular compartments involved in the morphogenesis and transport of new viral particles such as the Golgi apparatus and the membrane endoplasmic reticulum also requires energy that cannot totally be provided by host cell metabolism, perhaps particularly in harsher environments. A suggestion of a virus aiding thermal tolerance of a host has been made (Márquez et al., 2007). Therefore, the NCLDV auxiliary metabolic genes may be involved in energy generation by boosting, in either a dependent or independent manner, the energy metabolism enzymes of the host cell to create an appropriate environment for viral replication.

At this time, it is difficult to ascertain whether these viral primary metabolism enzymes function according to the exact mechanisms as their corresponding host orthologs. Further efforts are urgently required to elucidate whether these viral metabolism enzymes function independently. Such findings will help discern if the virus becomes autonomous and does not parasitize the metabolism of the host cell, or whether they are just enzymes that manipulate the host energetic metabolic system. Genetic and biochemical approaches must be considered going forward, including exploring individual viral enzymes functioning in a heterologous background, including complementation of knockout mutants (either in bacteria or yeast) to determine if the viral enzymes modulate host energy production.

The detection of the viral primary metabolic genes (with the potential to be involved in energy production) runs contrary to our traditional view of virus biology. Until recently, viruses have been regarded as parasites of the host cell’s energy machinery rather than encoding their own virus metabolic machinery (Moreira and López-García, 2009) and supporting the proposed virocell concept (Forterre, 2013). The notion of viral manipulation and expansion of the host metabolic network suggests that host cells with highest metabolic activity may be more permissive to viral infection. Metabolic modulation is thus the central hub of the host–virus dynamics. Further research in the coming years will help shed light on such unprecedented findings.
